# Comprehensive analysis of genome-wide DNA methylation across human polycystic ovary syndrome ovary granulosa cell

**DOI:** 10.18632/oncotarget.8544

**Published:** 2016-04-01

**Authors:** Jiawei Xu, Xiao Bao, Zhaofeng Peng, Linlin Wang, Linqing Du, Wenbin Niu, Yingpu Sun

**Affiliations:** ^1^ Center for Reproductive Medicine, The First Affiliated Hospital of Zhengzhou University, Zhengzhou, Henan 450000, China

**Keywords:** polycystic ovary syndrome, granulosa cell, DNA methylation, global DNA methylation and hydroxymethylation

## Abstract

Polycystic ovary syndrome (PCOS) affects approximately 7% of the reproductive-age women. A growing body of evidence indicated that epigenetic mechanisms contributed to the development of PCOS. The role of DNA modification in human PCOS ovary granulosa cell is still unknown in PCOS progression. Global DNA methylation and hydroxymethylation were detected between PCOS’ and controls’ granulosa cell. Genome-wide DNA methylation was profiled to investigate the putative function of DNA methylaiton. Selected genes expressions were analyzed between PCOS’ and controls’ granulosa cell. Our results showed that the granulosa cell global DNA methylation of PCOS patients was significant higher than the controls’. The global DNA hydroxymethylation showed low level and no statistical difference between PCOS and control. 6936 differentially methylated CpG sites were identified between control and PCOS-obesity. 12245 differential methylated CpG sites were detected between control and PCOS-nonobesity group. 5202 methylated CpG sites were significantly differential between PCOS-obesity and PCOS-nonobesity group. Our results showed that DNA methylation not hydroxymethylation altered genome-wide in PCOS granulosa cell. The different methylation genes were enriched in development protein, transcription factor activity, alternative splicing, sequence-specific DNA binding and embryonic morphogenesis. YWHAQ, NCF2, DHRS9 and SCNA were up-regulation in PCOS-obesity patients with no significance different between control and PCOS-nonobesity patients, which may be activated by lower DNA methylaiton. Global and genome-wide DNA methylation alteration may contribute to different genes expression and PCOS clinical pathology.

## INTRODUCTION

Polycystic ovary syndrome (PCOS) is a complex and highly heterogeneous women endocrine disorder, the features include ovarian dysfunction, menstrual disorders, hyperandrogenemia, insulin resistance, abdominal obesity and infertility [1]. The familial clustering and inheritance indicates genetic factors contributed to PCOS development [2, 3], in addition to genetic predisposition, environment and life style factors; emerging evidence suggests that epigenetic modification/regulation contributed to the development of PCOS [4, 5]. PCOS is the most prevalent diseases an affects about 7% of the reproductive-age women [6]. However, the etiology remains unclear.

DNA methylation(5mC) as one of the best-studied DNA modification is a major epigenetic modification of the genome generally inhibit gene expression, whereas DNA hydroxymethylation(5hmC) associated with increasing gene expression [7, 8]. Different level in 5mC at single specific loci can be sufficient to regulate gene expression [9]. However, 5mC changes at specific loci cannot indicate the changes occurring at global levels. In adult mammals, the level of 5hmC exhibit rather variable and show tissues and cell types specific [10, 11]. It had been reported that peripheral blood DNA global methylation was not significantly altered in PCOS compared with healthy controls [12]. However, whether global DNA methylaiton and hydroxymethylation involved in PCOS development or not was needed to explore.

Epigenetic changes as a potential mechanism causing PCOS development has been investigated in many studies before. It had reported that genome-wide DNA methylation profile of PCOS peripheral blood revealed that 79 differentially methylated genes between PCOS-insulin resistance and PCOS-noninsulin resistance patients, and 40 differentially methylated genes between PCOS patients and healthy controls [13]. Further study had detected significant differences genome-wide DNA methylation and expression patterns exist between PCOS ovaries and normal ovaries [14, 15]. These studies indicated that DNA methylation may in part be responsible for the PCOS phenotype. Previous study has revealed that gene body DNA methylation of ovarian granulosa cells associated genes expression in PCOS patients [16]. The granulosa cells of PCOS ovaries were variable sensitivity to FSH, show lower apoptotic rate and higher proliferation rate coupling with 211 genes differentially expressed [17–19]. Ovary genome-wide DNA methylation may mask specific cell DNA methyaltion pattern, such as follicular granulosa cells, which play key roles during oocyte development through intercellular communication [20, 21]. Hence, it is essential to genome-wide profile the different DNA methylation between PCOS patients and healthy controls granulosa cells.

To confirm epigenetic abnormality and to recover a novel mechanism for granulosa cells in PCOS development and follicular development, we systemly study the granulosa cell global DNA methylation and hydroxymethylation of both PCOS patients’ and healthy controls’, and genome-wide profile the DNA methylaiton of obesity and non-obesity PCOS patients’. Analysis of the epigenetic phenotypes and identification of specific epigenetic changes of granulosa cells will help us illustrate the epigenetic etiology of PCOS.

## RESULTS

### Basic clinical characteristics of study PCOS patients and controls

All the clinical characteristics of study participants were shown in Table 1. The obesity PCOS patients and non-obesity patients showed significant higher luteinizing hormone (LH), testosterone (T) and Basic Follicular Number compared with healthy controls. Obviously, the obesity PCOS patients had highest body mass index (BMI). There were no differences between the BMI of the non-obesity PCOS patients and healthy controls. Blood glucose concentration showed no significance among the obesity PCOS patients, non-obesity patients and healthy controls. During Controlled Ovarian Hyperstimulation of clinical treatment, the gonadotropin (Gn) does and day's treatment showed no significance. The free thyroxine 3 (FT3), free thyroxine 4 (FT4) and thyroid stimulating hormone (TSH) also showed no statistical significant among three groups. Ovarian granulosa cells from 24 patients were conducted to DNA methylation chip investigation and RT-qPCR.

**Table 1:** Clinical characteristic of PCOS patients and healthy controls

Basic Characteristic	PCOS-obesity	PCOS-nonobesity	Control	P value
Age	30.38±4.14	29.75±2.82	29.63±2.67	0.887
BMI(Kg/m^2^)	28.95±0.74	21.95±2.18	21.21±2.83	**3.43E-07**
FSH(IU/L)	5.64±1.78	5.92±1.25	6.55±1.69	0.51
E2(pg/ml)	51.52±28.19	43.63±25.33	43.58±18.65	0.758
P(ng/ml)	0.76±0.24	0.8±0.31	0.9±0.35	0.664
PRL(ng/ml)	12.06±6.45	14.79±8.47	20.8±8.38	0.0964
LH(IU/L)	7.5±3.34	9.54±5.16	4.28±1.47	**0.0285**
T(ng/ml)	0.45±0.12	0.64±0.26	0.24±0.11	**0.000976**
Basic Follicular Number	22.75±2.55	23.75±0.7	13.88±5.1	**8.54E-06**
Days of Gn	12.13±2.42	10.75±1.98	10.63±1.69	0.289
Dose of Gn	2248.44±934.47	1487.5±435.43	1651.56±484.14	0.0733
Blood Glucose(mmol/L)	5.06±0.48	4.67±0.36	4.75±0.38	0.161
FT3(pmol/L)	5.16±0.92	4.95±0.72	4.83±0.54	0.676
FT4(pmol/L)	11.05±1.49	10.52±1.15	11.23625±1.64	0.597
TSH(miu/L)	2±0.82	2.31±0.79	2.3275±0.93	0.687

### Global DNA methylation and hydroxymethylation status of PCOS patients and healthy controls granulosa cells

**Figure 1:**
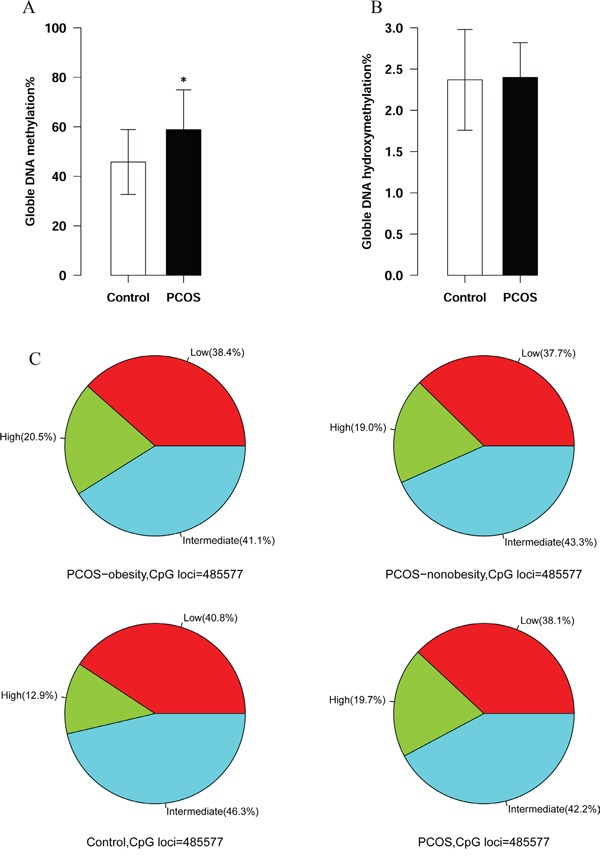
Global DNA methylation and hydroxymethylation between PCOS patients’ and Controls’ ovary granulosa cell **A.** Global DNA methylation of granulosa cell between PCOS patients and controls. The number of PCOS patients was 20 and controls were 20. P value <0.05 **B.** Global DNA hydroxymethylation of granulosa between PCOS patients and controls. The number of PCOS patients was 20 and controls were 20. P value >0.05 **C.** Hypermethylation CpGs distribution among PCOS-nonobesity, PCOS-obesity and Controls. (The hypermethylaiton of DNA methylation was defined lever above 0.8, the number of PCOS-obesity patients was 8, controls was 8 and PCOS-nonobesity was 8.)

Genome-wide global DNA methylation and hydroxymethylation may stimulate or activate gene expression activity. Although the global methylation of peripheral blood DNA was not significantly altered in PCOS compared with controls, it is still essential to investigation the global DNA methylation and hydroxymethylation in granulosa cells in PCOS [12]. In our study, the granulosa cell global DNA methylation of PCOS patients showed significant higher level than the controls’ (58.82 ± 16.1% VS. 45.76 ± 13.1%, P<0.05, Figure 1A). However, the global DNA hydroxymethylation was very low and showed no statistical difference between PCOS and control (2.40 ± 0.42% VS. 2.37±0.61%, P>0.05, Figure 1B). We further summarized the hypermethylation and hypomethylation in obesity PCOS patients, non-obesity patients and healthy controls. Our results showed that PCOS-obesity and PCOS-nonobesity patients harbored much more hypermethylated CpG sites (DNA methylation lever above 80%) than control (20.5% and 19.0% VS. 12.9%, Figure 1C), this further supported that granulosa cell DNA global methylation level of PCOS was higher than controls’.

### Genome-wide differentially DNA methylation genes profiling in PCOS

**Figure 2:**
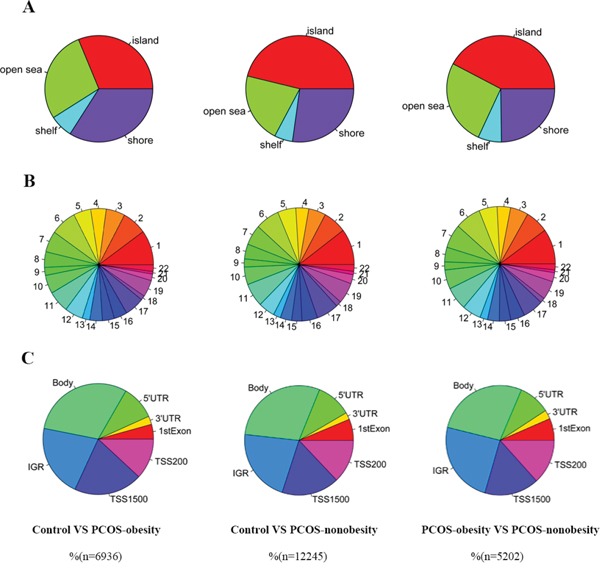
Different DNA methylation CpGs of granulose cells distribution in PCOS-obesity, PCOS-nonobesity and Control **A.** CpG content and neighbourhood context of CpG Island, shore, shelf and open sea distribution of different methylation CpG sites **B.** Different DNA methylation CpGs sites distribution across autosome (chromosome 1 to 22) **C.** Different DNA methylation CpGs sites gene structure: 5′UTR, 3′UTR, 1stExon, TSS200, TSS1500, IGR and gene body distribution. The number of PCOS-obesity patients was 8, controls were 8 and PCOS-nonobesity was 8.

Genome-wide DNA methylation analysis showed 6936 differentially methylated CpG sites (p<0.005) respectively between control and PCOS-obesity group and 2664 CpG sites exhibited highly significant (P<0.001, Supplementary Dataset 1A). There were 12245 differential methylated CpG sites (P<0.005) between control and PCOS-nonobesity group and 1582 CpG sites exhibited highly significant (P<0.001, Supplementary Dataset 1B). 5202 methylated CpG sites (P<0.005) were detected significantly differential between PCOS-obesity and PCOS-nonobesity group and 1186 CpG sites showed highly significant (P<0.001, Supplementary Dataset 1C). We further explored the different methylated CpG sites in methylated CpG content and neighborhood context (Shelf, Shore, Island and Open Sea); in particular, the different methylated sites between groups were residing in CpG islands (Control VS. PCOS-obesity: 31.17%, Control VS. PCOS-nonobesity: 46.14% and PCOS-obesity VS. PCOS-nonobesity: 43.76%, Figure 2A.) and Shore (Control VS. PCOS-obesity: 33.94%, Control VS. PCOS-nonobesity: 27.16% and PCOS-obesity VS. PCOS-nonobesity: 24.78%, Figure 2A), indicating that the gene expression may be activated or inactivated by DNA methylaiton located in CpG islands. However, no significant difference in methylated CpG sites residing in chromosomal locations was detected (Chr1-22 and XY) (Figure 2B). The different methylated CpG sites across the gene structure (TSS1500, TSS200, 5′UTR, First exon, Gene body, 3′UTR, and intergenic genomic region(IGR)) (Figure 2C), indicating the different methylated sites were located in gene boby and IGR, which would contributed to the gene expression alternative splicing. Overall, our data suggested the existence of a marked difference in methylation and regulation patterns, both of which are implicated in the development of PCOS. To further explore the different methylated sites whole genome distribution, we genome-wide display the different methylated sites across whole-genome (shown in Supplementary Figure 1, 2 and 3).

### Functional analysis of genome-wide different DNA methylated sites

**Figure 3:**
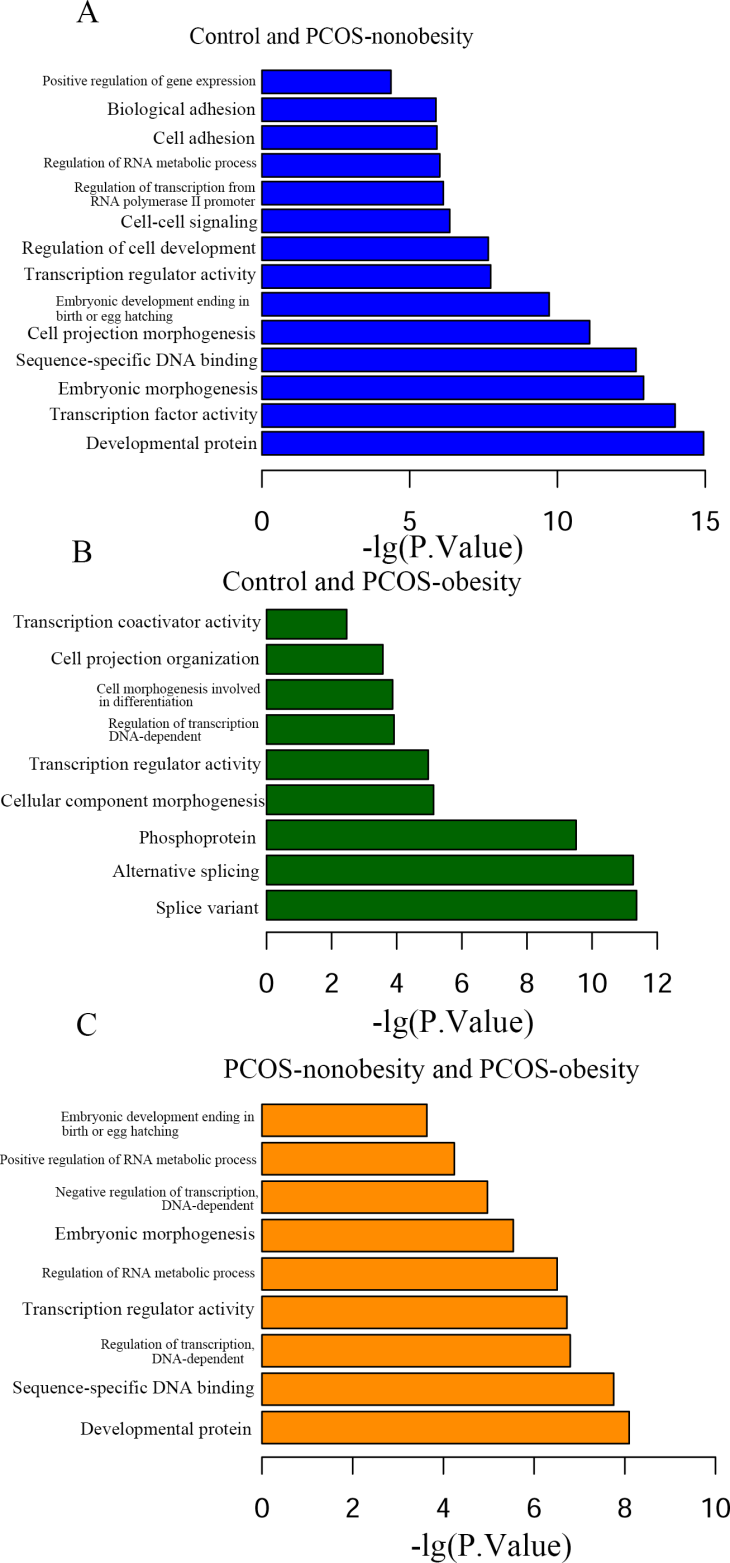
Functional analysis of different methylation sites of PCOS-obesity, PCOS-nonobesity and Control **A.** Different methylation sites between control and PCOS-nonobesity functional analysis using DAVID **B.** Functional analysis of different methylation sites between control and PCOS-obesity using DAVID **C.** Different methylation sites between PCOS-obesity and PCOS-nonobesity functional analysis using DAVID.

In order to explore the biological function of genes relevant in regulatory network of PCOS-obesity and PCOS-nonobesity, we further performed GO and KEGG analysis of the differentially methylated genes associated with PCOS-obesity and PCOS-nonobesity with DAVID. The all GO analysis results of three groups was shown in supplementary dataset 2 (Dataset 2A, Dataset 2B and Dataset 2C). The top significant GO terms associated with PCOS were shown in Figure 3. GO enrichment analysis for different DNA methylation sites of genes in GCs between Control and PCOS-nonobesity showed that development protein (False Discovery Rate (FDR) =1.64E-12), transcription factor activity (FDR=1.70E-11), embryonic morphogenesis (FDR=2.25E-10), sequence-specific DNA binding (FDR=3.52E-10) and embryonic development ending in birth or egg hatching (FDR=3.49E-07) were highly significant enriched. The GO categories splice variant (FDR=7.70E-09), alternative splicing (FDR= 7.99E-09), phosphoprotein (FDR= 4.53E-07) were the most significant enrichment between Control and PCOS-obesity. The GO categories developmental protein (FDR= 1.15E-05), sequence-specific DNA binding (FDR= 2.72E-05) and regulation of transcription DNA dependent (FDR=2.87E-04) were enrichment between PCOS-nonobesity and PCOS-obesity.

### Different DNA methylated site validation and target gene expression

**Figure 4:**
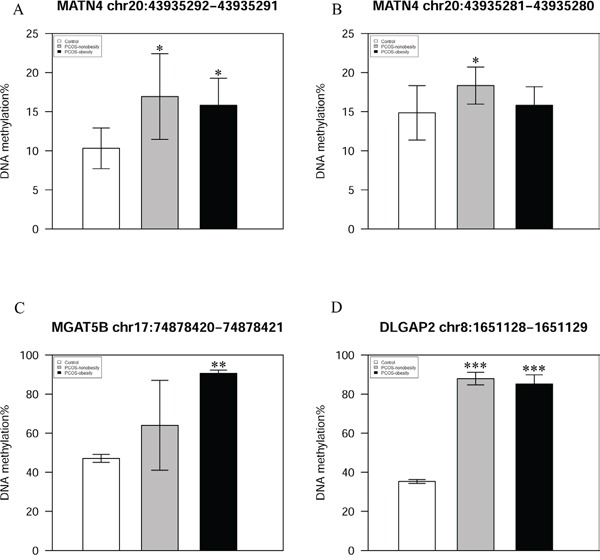
Validation of MATN, MGAT5B and DLGAP2 methylation site using pyrosequencing **A.** Validation of MATN4 Chr20:43935292-43935291 DNA methylation level **B.** DNA methylaiton level of MATN4 Chr20:43935281-43935280 **C.** Validation of MGAT5B chr17:74878420-74878421 DNA methylation level **D.** Validation of DLGAP2 chr8:1651128-1651129 DNA methylation level.

**Figure 5:**
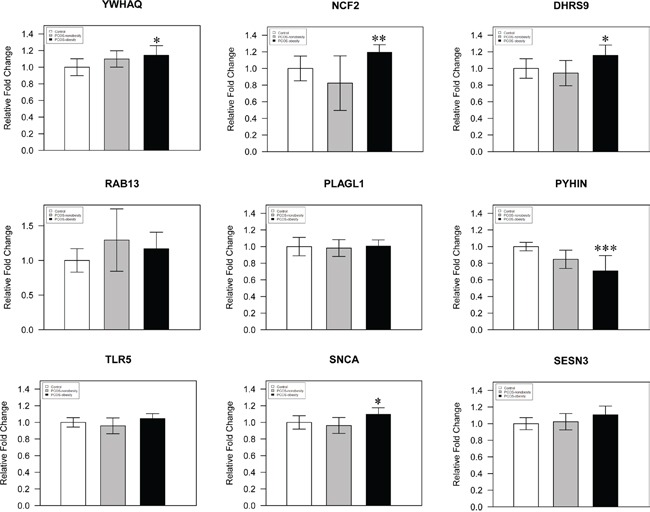
A histogram showing qPCR results of three groups for 9 selected genes The *YWHAQ, NCF2, DHRS9, RAB13, PLAGL1, PYHIN, TLR5, SNCA and SESN3* were analyzed by *Q-RT-PCR*, using total 24 samples from PCOS-obesity patients, controls and PCOS-nonobesity, each group 8 samples. *YWHAQ, DHRS9* and *SCNA* showed significantly higher in PCOS-obesity patients (P<0.05), *NCF2* showed highly significant up-regulation in PCOS-obesity groups(P<0.01), while PYHIN exhibited most significantly down-regulation in PCOS-obesity groups(P<0.001).

We used pyrosequencing to validate the methylated sites detected in our study. The cg27227742 of MATN4 (Chr20:43935292-43935291), cg21498547 of DLGAP2 (Chr8:1651128-1651129), and cg08292959 of MGAT5B (chr17:74878420-74878421) were selected in our study, and our pyrosequencing results was in consonance with our Infinium HumanMethylation 450 BeadChip data (Figure 4). In addition, we detected a site (MATN4 Chr20:43935281-43935280) showed higher level of methylation in PCOS-nonobesity than the other two groups (Figure 4B). DNA hypermethylation was known to down regulate gene expression, then Quantitative Real Time PCR was performed to evaluate gene expressions patterns of the nine genes previously reported different expressed in PCOS patients [19]. And our data showed that a different methylated site located in potential regulated region of those genes (Supplementary Table 2). We detected five genes were different expressed (Figure 5), *YWHAQ, NCF2, DHRS9* and *SCNA* only up-regulation in PCOS-obesity patients with no significance different between control and PCOS-nonobesity patients. However, *PYHIN* showed down-regulation in PCOS-obesity patients.

## DISCUSSION

PCOS affects approximately 7% of women of childbearing age, previous studies suggested that obesity plays a critical role in PCOS development and may reduce the pregnancy rate and results in cancellation rate during in vitro fertilization treatment [22]. Despite many factors have been proved play important role in PCOS development over the past decades, DNA modification in ovary granulosa cell remain unclear. In this study, we firstly system investigated the PCOS-obesity and PCOS-nonobesity ovary granulosa cell DNA modifications including global genome DNA methylation and DNA hydroxymehytlation, genome-wide DNA methylation and their potential regulation function on gene expression.

Obesity really showed obvious effects on both the development and the clinical manifestation of PCOS, mainly by increasing androgen availability, alternating function of granulosa cell and disturbing follicle development [23]. In our study, the PCOS-obesity patients showed higher BMI, LH and T compared with PCOS-nonobesity and control, which in accordance with previous studies [24]. Different gene expression patterns were detected in human PCOS granulose cell, indicating that PCOS profound affected the function of granulosa cell and may resulted in follicular development abnormal [19, 25, 26], the genetic and environmental roles in the development of the PCOS and dysregulation of gene expression remain unclear. Global DNA methylation and hydroxymethylation are key for maintaining tissue-specific gene expression regulation, indicated global DNA methylation and hydroxymethylation may be involved in gene activity [27]. Our results suggested higher global methylated DNA level in PCOS-obesity and PCOS-nonobesity patients, and harbored much more hypermethylated CpG sites, suggesting that genes expression were dysregulated in PCOS patients’ granulosa cell, DNA methylation may partlylead to gene expression alteration in PCOS patients granulose cell. However, global DNA hydroxymethylation level was very low in PCOS and control, and showed no significance, our data indicated that DNA methylation not hydroxymethylation may contribute to gene activity in PCOS ovary granulose cell. DNA hydroxymethylaiton was key epigenetic modification, may be involved in follicular development and oocyte maturation, but DNA hydroxymethylaiton showed low lever in human granulosa cell and may not contribute to PCOS progression. Hence, we concluded that DNA methylation alteration in human granulosa cell could contribute to obesity and non-obesity PCOS phenotype.

Methylation of the promoter region is well identified as a silencer; also the gene body has been proved as a regulator of gene expression in many tissues [28]. Our data showed that many different methylation sites were located in CpG islands and gene body, which indicated that these sites associated with gene expression. Functional enrichment analysis showed that the different methylated sites between three groups were related to development protein, splice variant, embryonic morphogenesis and transcription factor activity. Our global DNA methylation level and different methylated genes functional enrichment analysis indicated that DNA methylation was a key factor in development of PCOS and may affect follicular development and embryonic morphogeneis during IVF. Furthermore, our data showed that different methylated sites enriched in splice variant functional term, indicating that DNA methylation contributed to different genome-wide gene expression pattern between control and PCOS. We selected 9 genes which were shown different expression between PCOS insulin resistant and non-resistant previously published [19] and harbored different methylated sites, and 5 of them were observed different expressed potentially regulated by DNA methylation located in regulation region, which further support that DNA methylation contributed to different gene expression in granulosa cell between control and PCOS. *YWHAQ, NCF2* and *DHRS9* may be regulated by the different methylated sites, whereas there would other mechanism regulated *PYHIN* and *SCNA* expression. In our study, the selected previously 9 genes didn't show significant obvious difference between groups, ethnicity and markedly insulin resistance may affect the gene expression.

In summary, our current study discloses global DNA methylation, hydroxymethylation and specific DNA methylation patterns related to CpG Island, Open Sea, Shelf and Shore in the granulosa cells of PCOS obesity and nonobesity women. Considering these different DNA methylaiton genes are also linked to development protein, transcription factor activity, alternative splicing, sequence-specific DNA binding and embryonic morphogenesis, we concluded that these genes may be related to embryo development and metabolic disorders associated with the different clinical phenotypes of PCOS.

## MATERIALS AND METHODS

### Study patients recruitment

Totally 40 PCOS patients and 40 healthy controls were recruited from the Reproductive Center of the First Affiliated Hospital of Zhengzhou University (Zhengzhou, China). PCOS was defined by the revised Rotterdam diagnostic criteria for PCOS (Rotterdam ESHRE/ASRM-Sponsored PCOS Consensus Workshop Group, 2004). Other causes of oligomenorrhea or hyperandrogenism (for example, nonclassical 21-hydroxylase deficiency, Cushing's syndrome, hypothyroidism, significant elevations in serum prolactin) were excluded on clinical grounds in our study. The healthy control patients were those only with tubal blockage or male infertility coming for in vitro fertilization. Those patients were recruited based on the following criteria: (i) ≥25 and ≤35 years and (ii) normal karyotypes (46, XX) were shown in chromosome examinations for all of them. All our study was conducted in accordance with the ethical standards and according to the Helsinki Declaration of 1975. All patients were informed consents.

### Ovarian stimulation protocols, oocyte retrieval and granulosa cells (GCs) collection

All patients were using the GnRH agonist standard long protocol, ovarian stimulations were treated with recombinant FSH/HMG after pituitary down-regulation with GnRH agonist. The pituitary suppression was achieved with daily 0.1 mg triptorelin (Decapeptyl; Ferring Pharmaceutical, Kiel, Germany) from the mid-luteal phase of the cycle, after ovarian suppression was done; the dose of Decapeptyl was reduced to 0.05mg till the day of HCG administration. When the absence of dominant follicular development was confirmed sonographically and serum E2 < 30 mIU/mL, LH < 3 mIU/mL, recombinant FSH (Gonal-F, Serono, Switzerland) administration was initiated at daily dosages ranging from 150 to 300 IU, or in combination with hMG (hMG, Lizhu, China). Final follicular maturation was triggered by the administration of 4,000-10,000 IU human chorionic gonadotropin (hCG) when the largest follicles were more than 20 mm in diameter, as well as more than two thirds of the dominant follicles were greater than 16 mm in diameter. Transvaginal ultrasound-guided oocyte retrieval was scheduled 36-37h after HCG administration [29, 30].

The follicular fluid (FF) samples were carefully collected from the first aspiration follicle of each ovary, and only FF samples which did not contain any visible blood contamination were used in our study. The FF samples were then centrifuged for 15 min at 450g, and the supernatants were discarded. The granulose cells (GCs) were isolated from the blood cells and cellular debris using Percoll gradient centrifugation (Sigma) according to the user guide. The GCs were then for DNA and RNA extraction.

### Granulosa cell DNA/RNA extraction and global methylation/hydroxymethylation quantification

The GCs DNA and RNA were extracted using the AllPrep DNA/RNA Mini Kit (Qiagen, Hilden, Germany). All the manipulations were under the user guide. Global DNA methylation and hydroxymethylation status were detected using MethylFlash^TM^ Methylated and Hydroxymethylated DNA Quantification Kit (Epigentek, Epigentek Group Inc., USA), both of which are ELISA-like reactions. For methylated DNA status, according to the protocol, input DNA amount is 100 ng (18 samples, including 9 PCOS and 9 controls); while 200 ng for hydroxymethylation status detecting (17samples, including 9 PCOS and 8 controls). The absorbance was read on a microplate reader (Thermo MK3,) at 450 nm within 10 min after the color reaction. Global Methylation/Hydroxymethylation(%) = (OD (sample-blank) /2)/OD (positive control-blank) ×100%.

### Granulosa cell DNA bisulfite conversion and genome-wide DNA methylation profile using Illumina Infinium HumanMethylation 450 BeadChip

Bisulphite treatment of granulose cell DNA was performed using the EZ DNA CT Conversion Reagent following the manufacturer's protocols. A total of 1 μg of genomic DNA was used to bisulfite conversion using the EZ DNA Methylation Gold Kit (Zymo Research Corporation, Irvine, CA).

The genome-wide DNA methylation microarray screening was performed in 8 obesity-PCOS patients, 8 non-obesity-PCOS cases with normal BMI and 8 cases of control women. About 600 ng of the bisulfite-converted DNA was analyzed on Infinium HumanMethylation 450 BeadChip (Illumina, San Diego, CA) following the manufacturer's guidelines. These Chips feature more than 450,000 methylation sites within and outside CpG islands. Methylation values for individual CpG sites were obtained as β-values, calculated as the ratio of the methylated signal intensity to the sum of both methylated and unmethylated signals after background subtraction. Data were normalized to background intensity levels and displayed using Genome Studio software version 2010(Ilumina Inc.).

### Differential methylation genes screen and functional enrichment analysis

The overall different methylation loci were calculated using ChAMP [31] and Genome Studio software version 2010(Ilumina Inc.). DNA methylation profiling data were processed by the ChAMP package. Probes with a detection P>0.05 were removed, and β-values were compared between each two groups using two-sided Student's t-test. The p-value for differential methylation between two groups was calculated by limma package [32, 33]. Only P<0.05 were considered as statistical differences. GO analysis of differenti ally methylated genes between each two groups were performed using DAVID Bioinformatics Resources Version 6.7 according to previously published protocol [34, 35].

### Reverse transcription and real-time quantitative PCR

Total RNA of granulose cell was converted to cDNA with the QuantiTect Reverse Transcriptase Kit (Qiagen), using 1μg of total RNA and 1μl of random primer mix provided in the kit. Real-time PCR was performed using the 1μl cDNA in a total reaction volume of 20 μl containing 10 nM of forward and reverse primers each and 10 μl QuantiNova SYBR Green PCR Master Mix (Qiagen) and 0.1μl QN ROX Reference Dye (Qiagen). For each biological sample, Real-time PCR was performed in triplicate and the real-time quantitative PCR reaction were done on an ABI 7500 Sequence Detection System (Applied Biosystems; 40 cycles of 5 seconds of melting at 95°C followed by 30 seconds of annealing and extension at 60°C). 9 genes were quantitative analyzed in our study, the primers sequence could be obtained in Supplementary Table 1. The results were normalized using the β-actin as housekeeping gene. The data were analyzed using the ΔΔCt method.

### DNA methylation validation using pyrosequencing

To validation the different methylation sites of Infinium HumanMethylation 450 BeadChip, we used pyrosequencing reactions according to the manufacturer's instructions, using the PSQ 96 SNP Reagent Kit (Biotage AB). 3 CpG site located in *MATN4*, DLGAP2 and MGAT5B were selected in our study, the primer of MATN4 F was GTGGTAGAGAGTGGATTAAAATTTATT, the primer R was CCTACTAAACAAATATAAACTTCCCAC and the primer S was GTAGAGAGTGGATTAAAATTTATTG. The primer of DLGAP2 F was TTGTAGAGGGGTTGGGGATAT, the primer R was ACCCCAATACCTAATCTTCCTTCC and the primer S was GATATTGTAAAGTGTAAATTAAGG. The primer of MGAT5B F was TGGTTAGGTTGGAGAATAGTAGTGA, the primer R was ATACCACAATATAATCCAAATCTTCTC, and the primer S was GTGGGGAGGAGGTATA. The methylation level of each CpG site was analyzed by AQ Software. 8 samples were selected from the PCOS-obesity, PCOS-nonobesity and control groups.

### Statistical analysis

All the characters of PCOS patients and healthy controls were given as number of mean ± SD. The clinical characters were statistic by one-way-ANOVA. The global DNA methylation and hydroxymethlation was compared by Mann-Whitney *U* test. All probability values were two-sided, and P<0.05 was considered significant. * means P<0.05 and ** means P<0.01. *** means P<0.001. All the analyses were performed with R software (Version 3.2.2).

## SUPPLEMENTARY FIGURES AND TABLES










