# Supplementary material for: Comprehensive analysis of genome-wide DNA methylation across human polycystic ovary syndrome ovary granulosa cell
Source: Oncotarget. 2016 Apr 1;7(19):27899–909. doi: 10.18632/oncotarget.8544 (PMC5053696; doi:10.18632/oncotarget.8544)
Supplement: Supplementary file 1 [file oncotarget-07-27899-s001.pdf]

SUPPLEMENTARY FIGURES AND TABLES

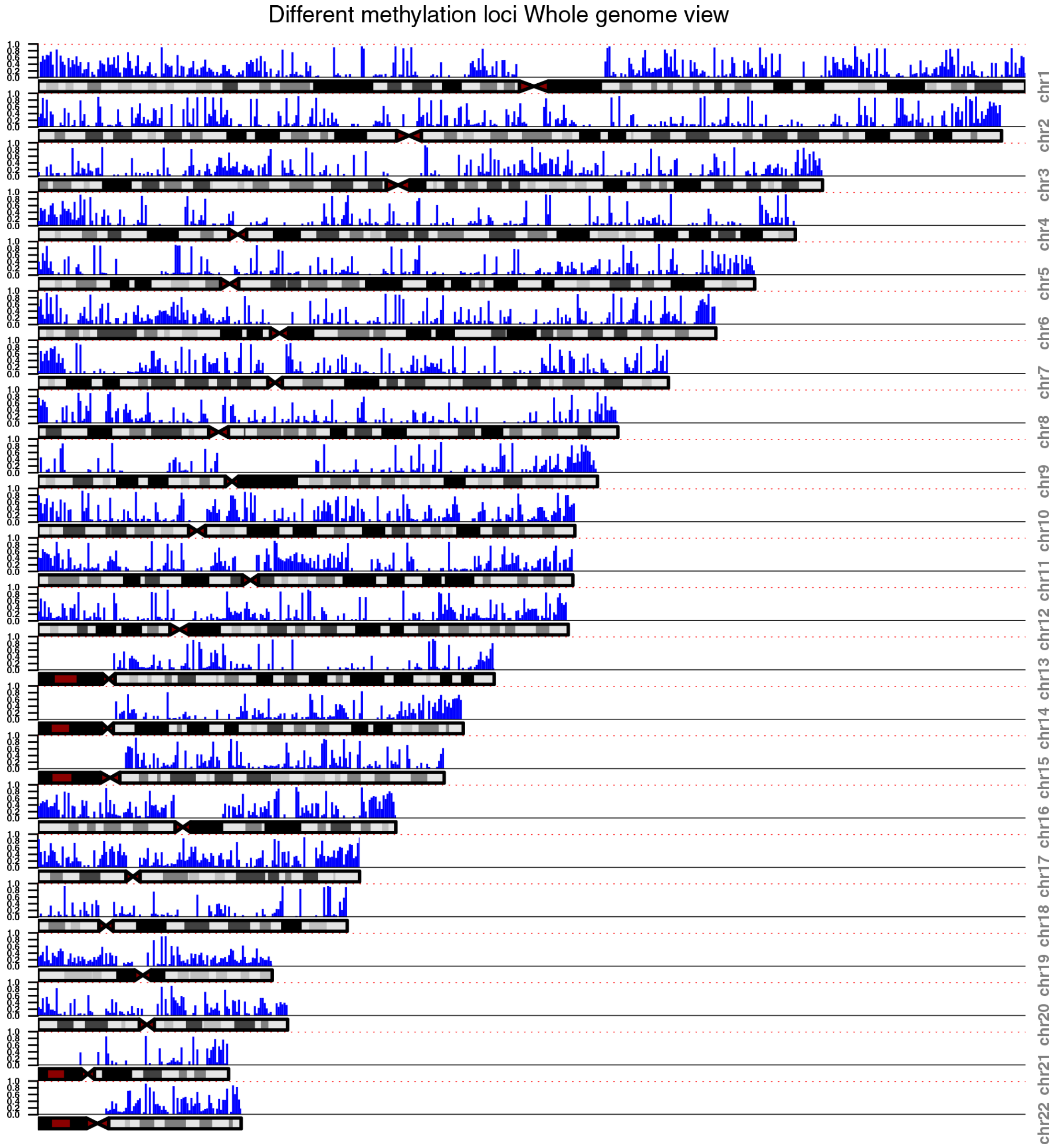

Supplementary Figure S1: Whole genome view of different methylation sites between control and PCOS-nonobesity.

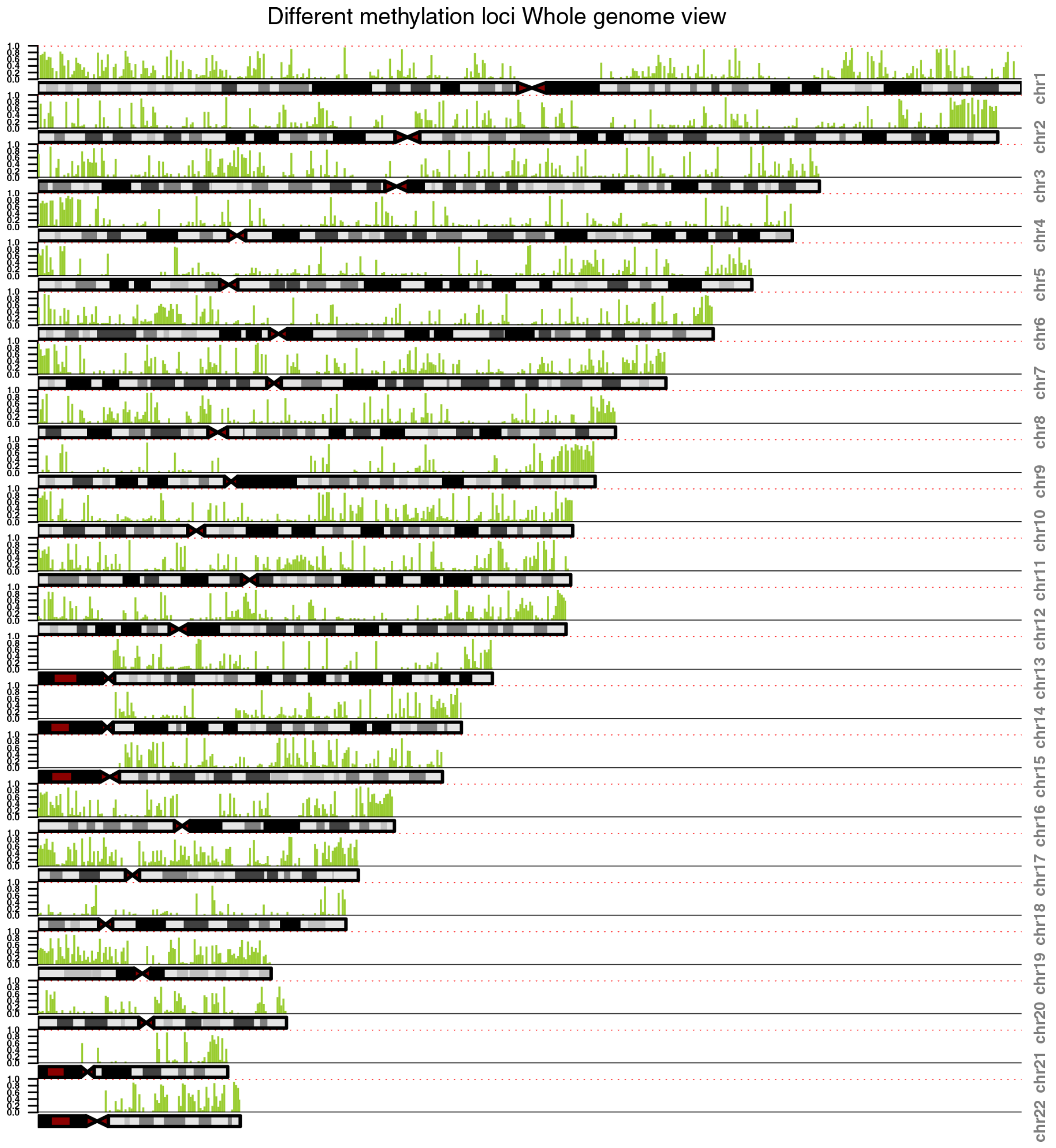

Supplementary Figure S2: Whole genome view of different methylation sites between control and PCOS-obesity.

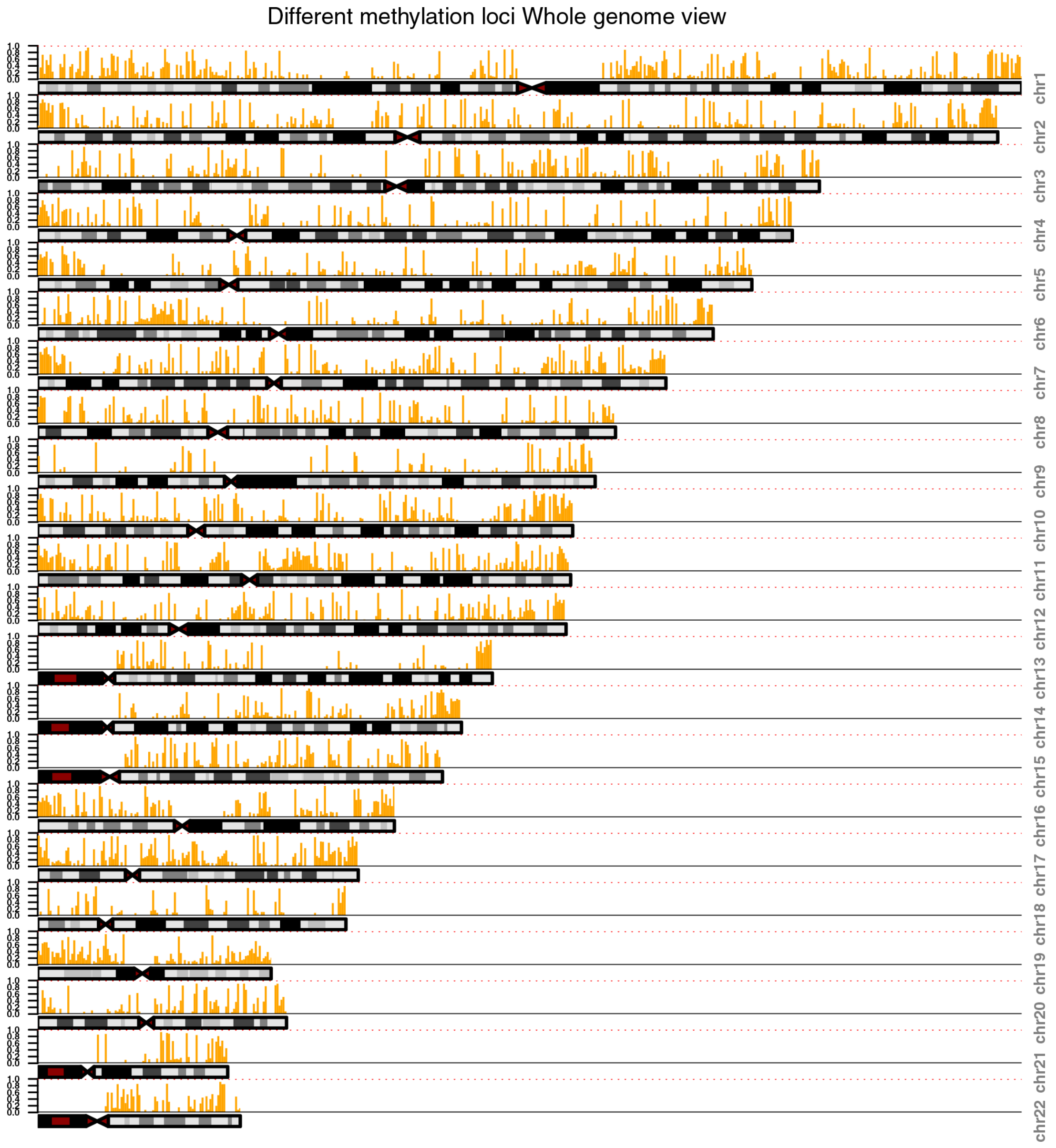

Supplementary Figure S3: Whole genome view of different methylation sites between PCOS-obesity and PCOS-nonobesity.

Supplementary Table S1: Real-time quantitative PCR primers of *YWHAQ, NCF2, DHRS9, RAB13, PLAGL1, PYHIN, TLR5, SNCA, SESN3*

See Supplementary File 1

Supplementary Table S2: Selected gene relative expression and Different methylated sites

See Supplementary File 2

Supplementary dataset 1. Different DNA methylation sites of three groups

Dataset 1A. List of significant DNA methylaiton sites of GCs between Control and PCOS obesity (P.Value <0.001)

Dataset 1B. List of significant DNA methylaiton sites of GCs between Control and PCOS non-obesity (P.value <0.001)

Dataset 1C. List of significant DNA methylaiton sites of GCs between PCOS non-obesity and PCOS obesity (P.value <0.001)

See Supplementary File 3

Supplementary dataset 2. Different DNA methylation loci functional enrichment analysis

Dataset 2A. GO enrichment analysis for different DNA methylation sites of genes in GCs between Control and PCOS-nonobesity (P.value < 0.05)

Dataset 2B. GO enrichment analysis for different DNA methylation sites of genes in GCs between Control and PCOS-obesity (P.value < 0.05)

Dataset 2C. GO enrichment analysis for different DNA methylation sites of genes in GCs between PCOS-nonobesity and PCOS-obesity (P.value < 0.05)

See Supplementary File 4
