# Supplementary material for: Comprehensive analysis of genome-wide DNA methylation across human polycystic ovary syndrome ovary granulosa cell
Source: Oncotarget. 2016 Apr 1;7(19):27899–909. doi: 10.18632/oncotarget.8544 (PMC5053696; doi:10.18632/oncotarget.8544)
Supplement: Supplementary file 2 [file oncotarget-07-27899-s002.pdf]

Supplementary Table 1. Real-time quantitative PCR primer used in our study

Primer	Sequence(5'to3')
PYHIN1-F	GCAACCGTCTCACAGCTAAAG
PYHIN1-R	AAGGCCGAGTCTGCTCTTTG
TLR5-F	GCCGGTCCTGTGTTTGGAAT
TLR5-R	CAGTGGTGTTGAGGACCTGG
SNCA-F	TGTGCGCTCCTTTTCCTTCT
SNCA-R	TGCTTCTCCACAACTCCGAC
YWHAQ-F	CCACGGTGCTGGAATTGTTG
YWHAQ-R	CTTCAGCAAGGTACCGGAAGT
NCF2-F	CAAGCTGTTTGCCTGTGAGG
NCF2-R	GGCTCAGACTTCATGCTCGT
DHRS9-F	GACCTCTCTCCGGTGGTAGA
DHRS9-R	TGCTGGCATGTGAGACAGAG
RAB13-F	GCATCAAGGAGAATGCCTCG
RAB13-R	TCTCGAGCCAACTTATCGGC
SESN3-F	TGACAAGAGGACCAAGTGCC
SESN3-R	TGATGTTGTCCAGACGACCG
PLAGL1-F	ACATCGCCAGTCGTGTGTG
PLAGL1-R	GAGACCTTGTACATCGTCAGG
