# Supplementary material for: Comprehensive analysis of genome-wide DNA methylation across human polycystic ovary syndrome ovary granulosa cell
Source: Oncotarget. 2016 Apr 1;7(19):27899–909. doi: 10.18632/oncotarget.8544 (PMC5053696; doi:10.18632/oncotarget.8544)
Supplement: Supplementary file 3 [file oncotarget-07-27899-s003.pdf]

Supplementary Table 2. Selected gene relative expression and Different methylated sites

Control and PCOS-nonobesity

Gene	Fold change#	P.Value	CHR	MAPINFO	Feat.cgi	Control_AVG	PCOS-nonobesity_AVG	deltaBeta
PYHIN1	8.11	0.006489178	1	158900384	TSS1500 - open sea	0.762055082	0.687066165	-0.074988917
TLR5	2.11	0.000422424	1	223283205	3'UTR - open sea	0.877316353	0.829423139	-0.047893213
SNCA	-5.02	0.001646485	4	90759203	TSS1500 - shore	0.059863183	0.080094262	0.020231079
		0.002333543	4	90757351	5'UTR - shore	0.183764333	0.252562064	0.068797731
		0.007981682	4	90758797	TSS1500 - island	0.041721435	0.053530271	0.011808836
		0.008177568	4	90758120	5'UTR - island	0.06171539	0.079524281	0.01780889
		0.008359237	4	90758537	TSS1500 - island	0.041901636	0.05575203	0.013850395

Control and PCOS-obesity

Gene	Fold change#	P.Value	CHR	MAPINFO	Feat.cgi	Control_AVG	PCOS_obesity_AVG	deltaBeta
NCF2	2.03	0.003678526	1	183552095	Body - open sea	0.87398824	0.817383526	-0.056604714
DHRS9	-2.39	0.007962707	2	169945470	Body - open sea	0.667993666	0.740390271	0.072396605
YWHAQ	-2.13	0.26195363	2	9734163	Body - open sea	0.839632389	0.790595051	-0.049037338
RAB13	-1.6	0.0010549	1	153958977	TSS200 - open sea	0.102770469	0.145495135	0.042724666

PCOS-nonobesity and PCOS-obesity

Gene	Fold change#	P.Value	CHR	MAPINFO	Feat.cgi	PCOS-nonobesity_AVG	PCOS-obesity_AVG	deltaBeta
SESN3	12.98	0.006611877	11	94964345	TSS200 - island	0.147305187	0.12281077	-0.024494417
PLAGL1	2.75	0.001659102	6	144385609	1stExon - island	0.172175559	0.149875576	-0.022299983

#Fold change data were got form previously published data[1]

1 Kaur, S. *et al.* Differential gene expression in granulosa cells from polycystic ovary syndrome patients with and without insulin resistance: identification of susceptibility gene sets through network analysis. *The Journal of clinical endocrinology and metabolism* **97**, E2016-2021, doi:10.1210/jc.2011-3441 (2012).
